# HtrA3 as an Early Marker for Preeclampsia: Specific Monoclonal Antibodies and Sensitive High-Throughput Assays for Serum Screening

**DOI:** 10.1371/journal.pone.0045956

**Published:** 2012-09-25

**Authors:** Kemperly Dynon, Sophea Heng, Michelle Puryer, Ying Li, Kelly Walton, Yaeta Endo, Guiying Nie

**Affiliations:** 1 Prince Henry’s Institute of Medical Research, Clayton, Victoria, Australia; 2 Cell-Free Science and Technology Research Centre, Ehime University, Matsuyama, Japan; 3 Department of Biochemistry and Molecular Biology, Monash University, Clayton, Victoria, Australia; State Key Laboratory of Reproductive Biology, Institute of Zoology, Chinese Academy of Sciences, China

## Abstract

Mammalian HtrA3 (high temperature requirement A3) is a serine protease of the HtrA family. It has two isoforms [long (HtrA3-L) and short (HtrA3-S)] and is important for placental development and cancer progression. Recently, HtrA3 was identified as a potential diagnostic marker for early detection of preeclampsia, a life-threatening pregnancy-specific disorder. Currently there are no high-throughput assays available to detect HtrA3 in human serum. In this study we generated and fully tested a panel of five HtrA3 mouse monoclonal antibodies (mAbs). Three mAbs recognised both HtrA3-L and HtrA3-S and the other two detected HtrA3-L only. All five mAbs were highly specific to HtrA3 and applicable in western blotting and immunohistochemical analysis of endogenous HtrA3 proteins in the mouse and human tissues. Amplified luminescent proximity homogeneous assays-linked immunosorbent assays (AlphaLISAs), were developed to detect HtrA3 isoforms in picomolar levels in serum. The HtrA3 AlphaLISA detected significantly higher serum levels of HtrA3 in women at 13–14 weeks of gestation who subsequently developed preeclampsia compared to gestational-age matched controls. These HtrA3 mAbs are valuable for the development of immunoassays and characterisation of HtrA3 isoform-specific biology. The newly developed HtrA3 AlphaLISA assays are suitable for large scale screening of human serum.

## Introduction

The high temperature requirement A (HtrA) proteases are a well conserved family of serine proteases identified in organisms ranging from bacteria to mammals [1]. HtrAs are known to have important functions in protecting cells from stress conditions such as heat shock, oxidative stress, inflammation, ischemia/reperfusion and cancer [2].

To date, there are four mammalian HtrA homologues identified [1]. The first three members (HtrA1, HtrA2/Omi, HtrA3) have been cloned and investigated for expression and function [3]. The fourth HtrA (HtrA4) has only recently been characterised [2], [4].

HtrA3 was initially identified in the developing placenta both in the mouse and human as a serine protease associated with pregnancy [5]–[8]. HtrA3 is now known to inhibit trophoblast invasion during placental development [9], [10], and regulate ovarian development, granulosa cell differentiation and luteinisation [11], [12]. Studies in mice have also suggested that HtrA3 inhibits TGF-β signalling during embryo development [13].

**Figure 1:**
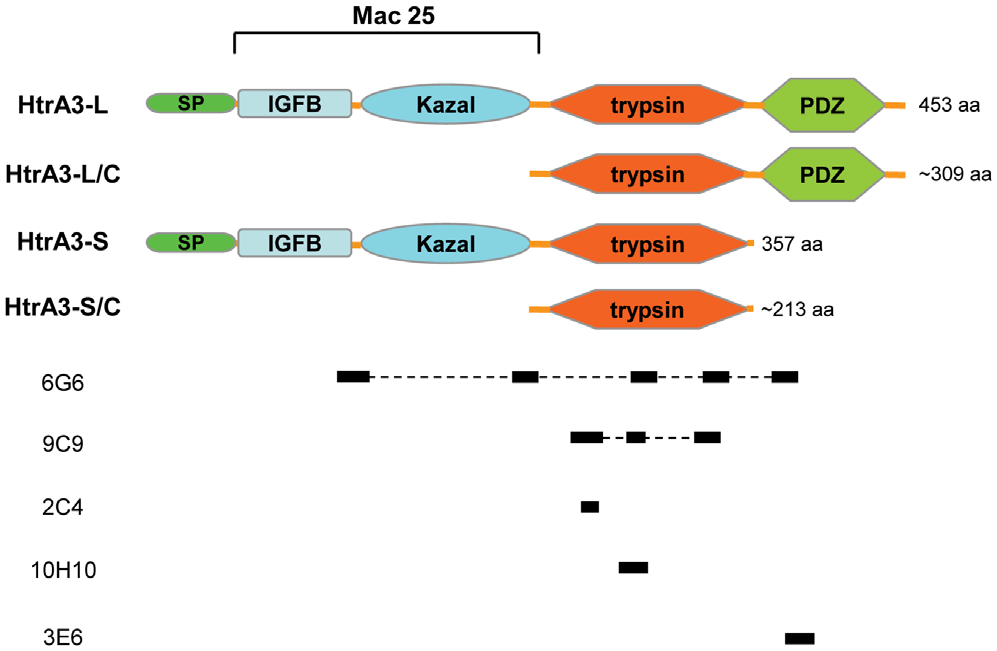
Schematic representation of HtrA3 protein domains and mAb epitope locations. The domain structures of human HtrA3-L and HtrA3-S proteins and their putative cleaved products HtrA3-L/C and HtrA3-S/C are shown. The Mac25 region is indicated. The solid bars below the protein domains denote the epitope location of each mAb, the four mAbs 6G6, 9C9, 10H10 and 3E6 were raised against HtrA3-L-S305A protein, and 2C4 was raised against a synthetic peptide. SP, signal peptide; IGFB, IGF-binding domain; Kazal, Kazal-type S protease inhibitor domain; trypsin, trypsin-like protease domain; PDZ, PDZ domain.

HtrA3 has two isoforms [long (HtrA3-L) and short (HtrA3-S)] resulting from alternative mRNA splicing [5], [6] (Figure 1). Full length human HtrA3-L and HtrA3-S contain 453 amino acids (aa) and 357 aa respectively. Both isoforms contain an N-terminal insulin-like growth factor binding (IGFB) domain and a Kazal protease-inhibitor domain followed by a signature trypsin-like serine protease domain. The HtrA3-L isoform differs from HtrA3-S with the presence of a C-terminal PDZ (post-synaptic density 95, *Drosophila* discs large, zona-occludens 1) domain (Figure 1) [5], [6]. HtrA3-S thus presents a naturally occurring HtrA lacking the C-terminal PDZ domain. Together, the N-terminal IGFB and Kazal domains in HtrA3 form a region homologous to Mac25, a follistatin-like protein with activin binding properties [14] (Figure 1). HtrA3 proteins undergo auto-cleavage. Protease dependent auto-catalytic cleavage of the Mac25 region from HtrA3 enhances protease activity and is necessary for mitochondrial to cytoplasmic translocation of HtrA3 and increased cell death [13], [15].

The HtrA3 protein shares 95% similarity between mice and humans [5], [6], and is expressed more abundantly in the heart, ovary, testis and placenta than in other tissues in the mouse [6]. While HtrA3-L is the predominant isoform expressed in the mouse [6], both HtrA3-L and HtrA3-S are expressed in human tissues, especially in the placenta [7]. It is unknown whether HtrA3-L and HtrA3-S are biochemically distinct, but the PDZ domain in HtrA3-L is predicted to regulate substrate specificity and possibly cellular localisation [5].

Dysregulation of HtrA3 is associated with the development of a number of diseases including cancer and preeclampsia [15]–[22]. HtrA3 is downregulated in lung cancers through smoking-induced DNA methylation and this downregulation increases cancer cell longevity [16]. HtrA3 downregulation in lung cancer also contributes to resistance to chemotherapeutic treatments such as etoposide and cisplatin [15].

HtrA3 is reported to inhibit TGF-β signalling in the endometrium [20], and is suggested to be involved in ovarian cell homeostasis and tumourigenesis [19]. Downregulation of HtrA3 is associated with the progression of endometrial and ovarian cancer [17]–[19]. HtrA3 is thus proposed to be a tumour suppressor and a potential therapeutic aid in cancer treatment [3], [15].

In contrast, sustained expression of HtrA3 in the first trimester of pregnancy is associated with the development of preeclampsia [22], [23]. Preeclampsia is a pregnancy-specific multisystemic disorder primarily involving hypertension and proteinuria. Preeclampsia occurs in 2–10% of pregnancies worldwide [24], and is responsible for 14% of pregnancy related mortalities [25]. Women in developing countries with reduced access to medical treatment in particular, face a higher incidence (up to 16.7%) of preeclampsia-related morbidity and mortality compared to those in developed countries [24].

Preeclampsia occurs after 20 weeks of gestation in previously normotensive women. Abnormal first trimester placentation generates a high state of maternal inflammation, preceding the onset of preeclampsia. Preeclampsia often restricts blood flow to the foetus causing intrauterine growth restriction. As symptoms of preeclampsia only resolve once the placenta is removed, the present “cure” for preeclampsia is delivery of the baby and the placenta, sometimes prematurely. Aside from complications resulting from premature birth, babies born from a preeclamptic pregnancy have a higher risk of developing chronic diseases (endocrine, nutritional and metabolic diseases, as well as stroke and hypertension) in adulthood [26], [27]. Women who have had a preeclamptic pregnancy also have a higher risk for cardiovascular disease later in life [28].

Early diagnosis of preeclampsia is critical for timely clinical intervention and supportive management of pregnant mothers and their foetuses. However, to date there is no clinically useful biochemical diagnostic assay that can predict preeclampsia in early pregnancy. As persistently high serum levels of HtrA3 are detected at the end of the first trimester in pregnant women who subsequently develop preeclampsia [22], we have proposed that monitoring HtrA3 in maternal blood during early pregnancy would identify women at risk for preeclampsia.

In this study we describe the development and characterisation of a panel of highly specific HtrA3 monoclonal antibodies (mAbs). These mAbs were epitope-mapped and tested for a range of applications including western blotting and immunohistochemistry (IHC). Importantly, the mAbs were used to develop highly sensitive and high-throughput Amplified luminescent proximity homogeneous assay-linked immunosorbent assays (AlphaLISAs), based on luminescent oxygen channelling immunoassay technology, to detect HtrA3 in human serum. After being validated on recombinant HtrA3 proteins, the optimised assays were applied to first trimester serum samples collected from women who subsequently developed preeclampsia later in pregnancy. Significantly higher levels of HtrA3 were detected in preeclamptic cases compared to gestational-age matched controls, consistent with previous reports. This result demonstrates the critical importance of these HtrA3 mAbs and the newly developed HtrA3 serum detection assays for the diagnosis of preeclampsia and other diseases associated with HtrA3 dysregulation.

## Materials and Methods

### Ethics Statement

#### Human ethics

Written informed consent was obtained from all participants, and ethical approval was obtained from the Southern Health Human Research Ethics Committee, Melbourne, Australia (Ethics approval numbers 04141C and 06014C).

**Table 1:** Characteristics of Preeclampsia and Control Subjects.

	Preeclampsia (n = 7)	Control (n = 8)
**Maternal age (y)**	32.5±1.5	31.87±0.97
**Preterm Preeclampsia**		
**(Diagnosis at <37 weeks)**	2 (28.5%)	–
**Term Preeclampsia**		
**(Diagnosis at >37 weeks)**	5 (71.5%)	–
**Gestational week at delivery**	38.87±0.6	>39
**Mode of delivery**		
**Normal vaginal delivery**	4 (57%)	8 (100%)
**Emergency caesarean section**	3 (42.8%)	–
**Live Birth**	7 (100%)	8 (100%)
**Birth weight centile**		
**>90th centile**	3 (42.8%)	8 (100%)
**50th centile**	2 (28.6%)	
**30th centile**	1 (14.3%)	
**20th centile**	1 (14.3%)	

Samples were collected at Monash Medical Centre, Melbourne, Australia [22].

#### Animal ethics

Mice were housed and handled according to Monash University Animal Ethics guidelines on the care and use of laboratory animals. All studies were approved by the Animal Ethics Committee at Monash Medical Centre, Melbourne, Australia (Ethics approval number 2005/25).

**Figure 2:**
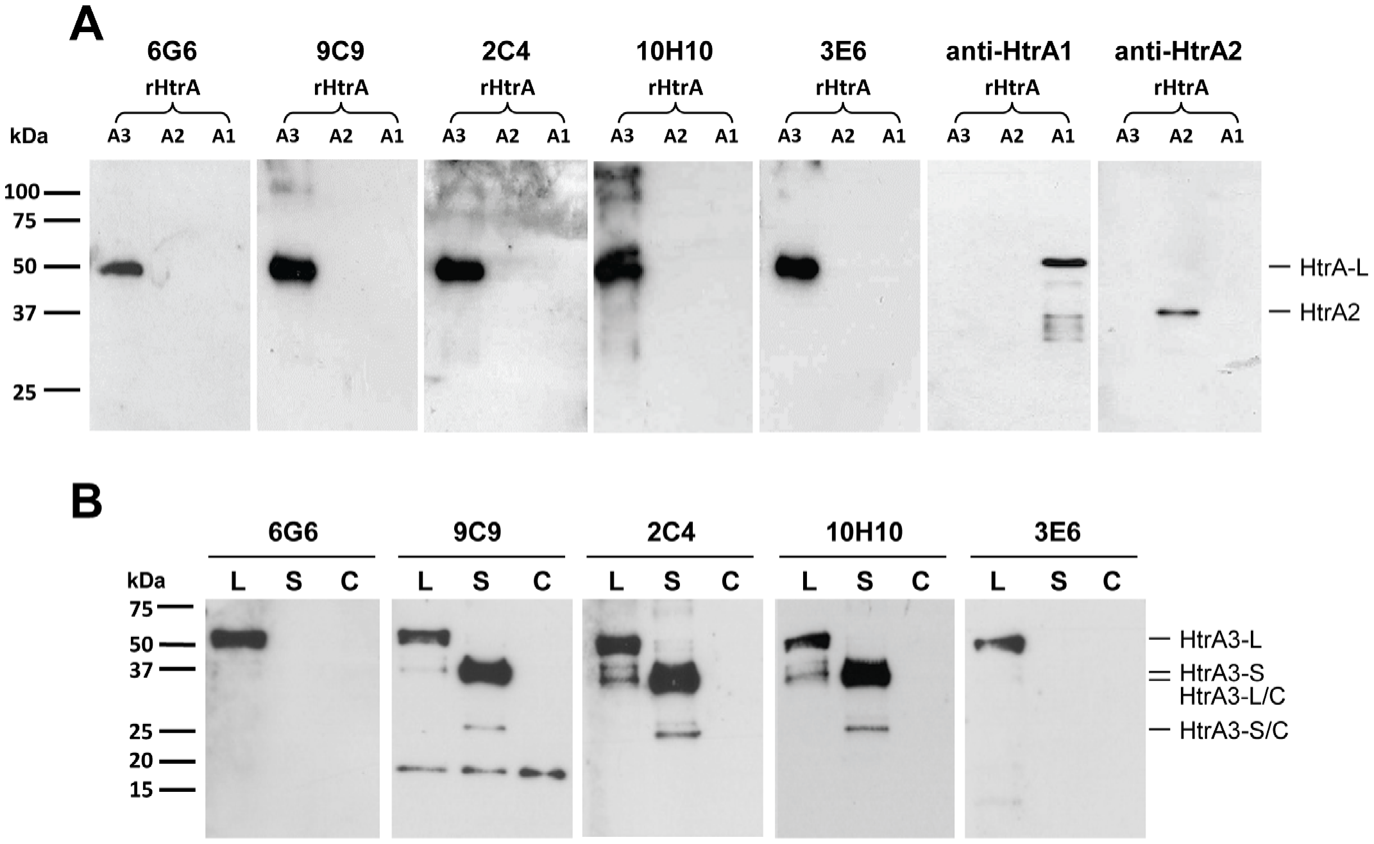
Confirmation of HtrA3 mAb specificity. (**A**) An equal amount (50 ng) of recombinant (r) human HtrA proteins [rHtrA1 (A1), rHtrA2 (A2) and rHtrA3 (A3, HtrA3-L-S305A)] were separated on reducing 12% SDS-PAGE gels and analysed using western blotting with HtrA3 mAbs (6G6, 9C9, 2C4, 10H10 and 3E6), anti-HtrA1 and anti-HtrA2 antibodies respectively. (**B**) Western blot analysis of neat conditioned media from HEK-293F cells transfected with control plasmids (control transfection, C) or WT HtrA3-L (L) or HtrA3-S (S) constructs with the five HtrA3 mAbs. The cleaved forms of HtrA3 are indicated as HtrA3-L/C and HtrA3-S/C.

### Recombinant Human HtrA Proteins

C-terminally His-tagged full length HtrA1 protein (produced in insect cells) and mature HtrA2 protein (produced in *Escherichia coli)* were purchased from Creative Biomart (Shirley, NY, USA). C-terminally His-tagged human wild type (WT) HtrA3-L produced in insect cells was purchased from ProteaImmun GmbH (Berlin, Germany). C-terminally His-tagged catalytically-inactive HtrA3-L (HtrA3-L-S305A, where serine residue 305 in the catalytic site was substituted with alanine) was synthesised using the wheat germ cell free technology as previously described [29] and purified using Ni-NTA agarose (QIAGEN, GmbH, Hilden, Germany).

**Figure 3:**
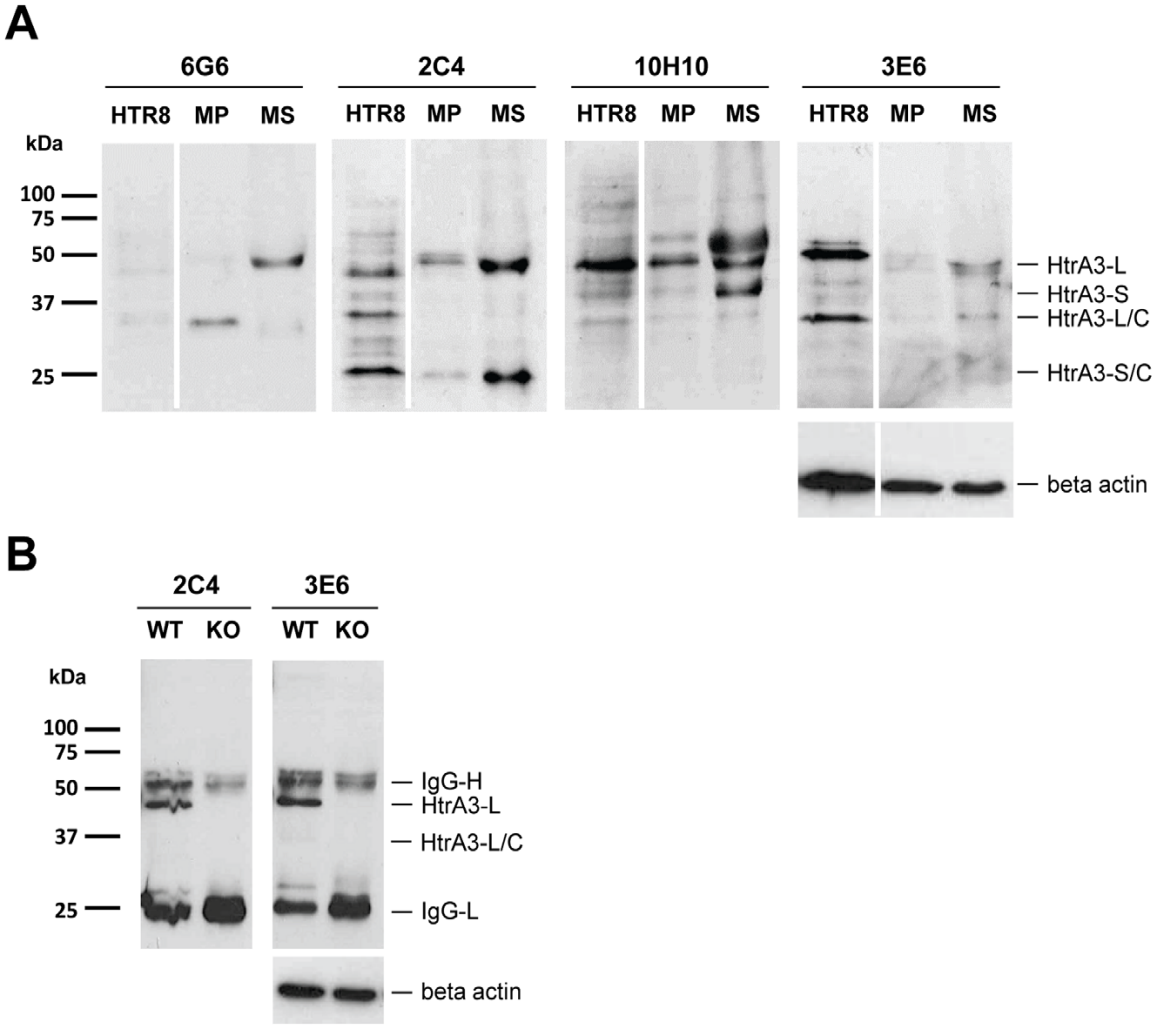
Western blot analysis of protein lysates from human and mouse reproductive tissues using HtrA3 mAbs. Cell lysates (50 µg) and tissue lysates (25 µg) were separated on reducing 12% SDS-PAGE gels. β-actin served as a loading control. (**A**) Lysates from human trophoblast cell line HTR8 (HTR8) and human endometrial tissues of the mid-proliferative (MP) and mid-secretory (MS) phases of the menstrual cycle were probed with mAbs 6G6, 2C4, 10H10 and 3E6. HtrA3-L and HtrA3-S and their respective cleaved forms HtrA3-L/C and HtrA3-S/C are indicated. (**B**) Lysates from embryo implantation sites on d10.5 of pregnancy from WT or HtrA3 knockout (KO) mice were probed with mAbs 2C4 and 3E6.

### HtrA3 mAb Production

HtrA3 mAbs were produced at the antibody facility at The Walter and Eliza Hall Institute of Medical Research, Bundoora, Victoria, Australia, using standard protocols of the facility. In brief, BALB/c mice were injected intraperitoneally with 50 µg of HtrA3-L-S305A protein or 30 µg of a synthetic peptide (TIKIHPKKKL, corresponding to aa 230–239 of both HtrA3-L and HtrA3-S, and conjugated to Keyhole Limpet Hemocyanin (KLH) through a C-terminal Cys; Mimotopes, Victoria, Australia). The mice received two additional injections of the same antigen/dose at the same site at four and eight weeks later. Ten days after the third immunisation, the mice were bled and the sera was screened by ELISA on HtrA3-L-S305A protein or the peptide (without KLH conjugation) to determine antibody titre. The sera were also screened using western blotting on HtrA3-L-S305A protein. The mice were rested for four weeks, and the highest respondent mouse in each immunisation group was selected for a final booster immunisation. Four days later, mice were sacrificed and blood and spleens were collected. A single cell suspension of spleen cells was prepared, washed in serum-free Dulbecco’s modified Eagle’s medium (DMEM, Invitrogen, Life Technologies Australia Pty Ltd, Mulgrave, Victoria, Australia), and fused with Sp2/0 myeloma cells in the presence of 50% (w/v) polyethylene glycol 1500. The cells were resuspended in Hybridoma Serum Free Medium (Invitrogen), supplemented with 10% (v/v) FCS, HAT (hypoxanthine, aminopterin, and thymidine) medium (Invitrogen) and IL-6 (made in-house from P388D1 cell line, used at 1∶1000 dilution). Cell suspensions were seeded in 96-well Falcon microwell flat-bottom plates (2×10^5^ cells/well) and selected in HAT medium by overnight culture. After 10–12 days, the supernatants from hybridoma clones were harvested and screened by ELISA on HtrA3-L-S305A protein or the peptide. The positive hybridomas were further confirmed on HtrA3-L-S305A protein using western blotting. Individual positive hybridomas were subcloned by standard limit dilution and screened using ELISA and then western blotting as specified above.

**Figure 4:**
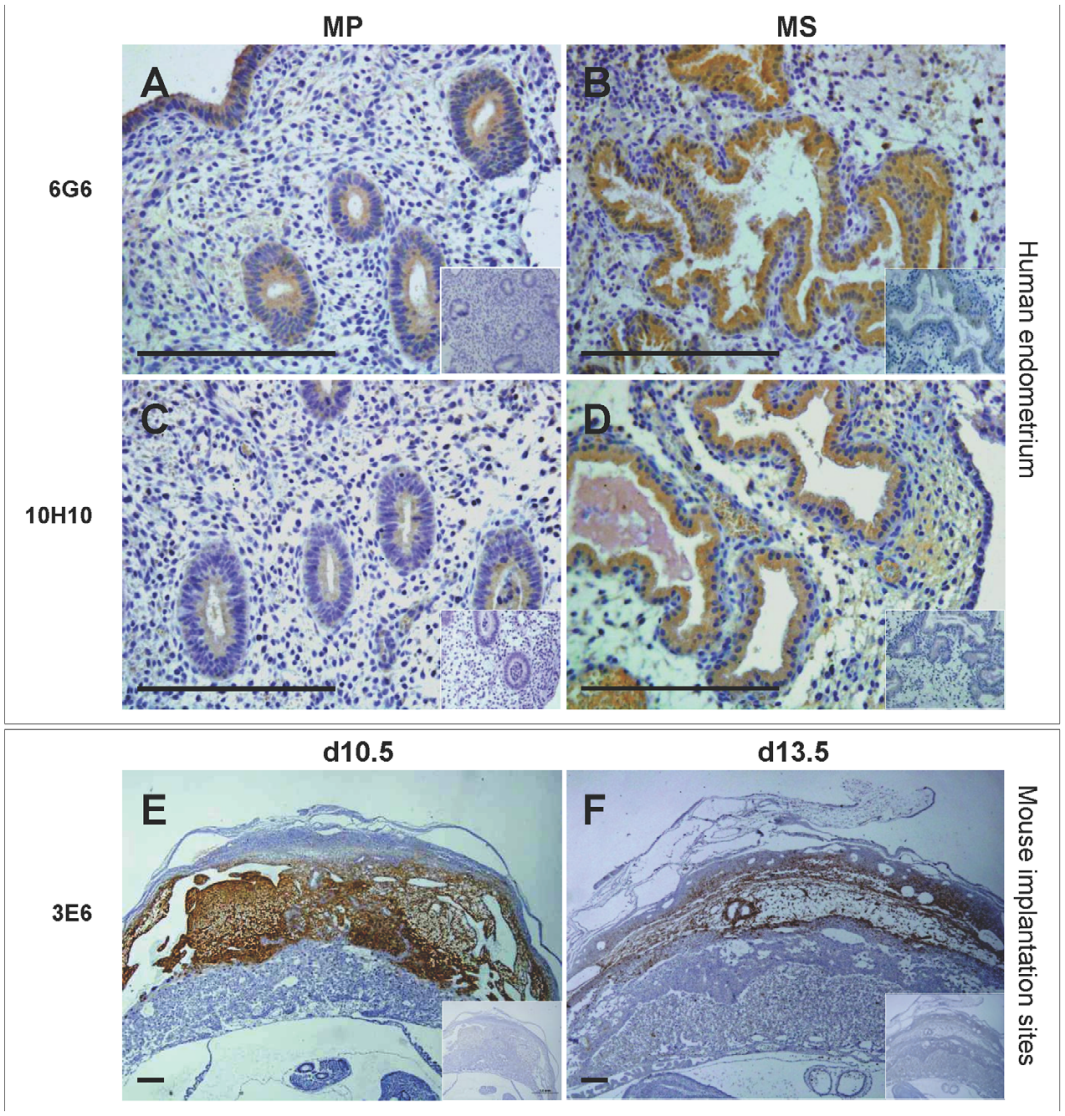
Immunohistochemical analysis of HtrA3 proteins in human and mouse reproductive tissues using HtrA3 mAbs. Representative images of immunostaining in human endometrial tissues from the mid-proliferative (MP, A and C) and mid-secretory (MS, B and D) phases of the menstrual cycle with mAbs 6G6 (A and B) and 10H10 (C and D). Representative images of immunostaining in mouse embryo implantation sites on d10.5 (E) and d13.5 (F) of pregnancy with mAb 3E6.

HtrA3 mAbs were purified from clonal hybridoma supernatants using Protein A or G Sepharose columns, eluted into sterile PBS, isotyped and retested on HtrA3-L-S305A protein using western blotting. Purified mAbs were aliquoted and stored at −20°C for long term storage or at 4°C for frequent use.

**Figure 5:**
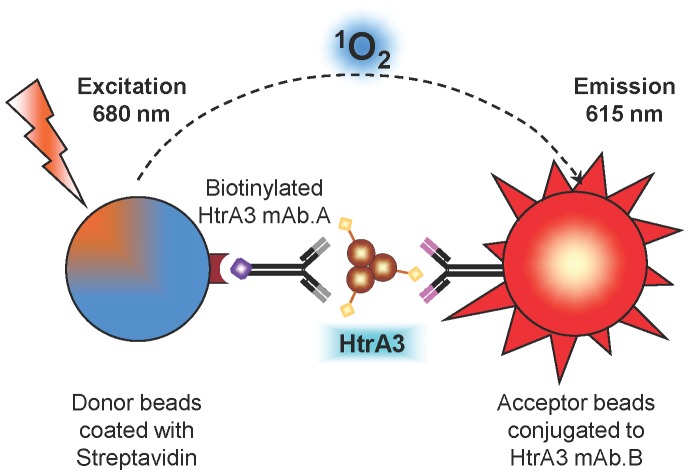
Schematic illustration of HtrA3 AlphaLISA principle. The AlphaLISA assay requires two HtrA3 antibodies recognising non-overlapping epitopes, one antibody is biotinylated and the other conjugated to acceptor beads. In the presence of HtrA3 proteins, the streptavidin-coated donor beads (blue) are brought into close proximity to the anti-HtrA3-coated acceptor beads (red). When excited at 680 nm, the Alpha Donor beads release singlet oxygen (_Δ_^1^O_2_) which travels to the nearby Acceptor beads and induces a chemiluminescent emission at 615 nm, the AlphaLISA signal.

### Epitope Mapping of mAbs

The linear or continuous epitopes of the mAbs raised against HtrA3-L-S305A protein were determined by screening a custom-synthesised peptide library (PepSet, Mimotopes). The full length amino acid sequence of human HtrA3-L (minus the signal peptide) was used to synthesise a complete set of 86 overlapping 15-aa peptides (overlap by 10 and offset by 5 aa). The N-terminus of each of the 86 peptides was conjugated to biotin followed by a 4-aa spacer SGSG and the C-terminus was amidated. The PepSet peptides were immobilised on streptavidin-coated/BSA-blocked 96-well plates (Pierce, Rockford, IL USA) and used for screening hybridoma supernatants using standard ELISAs. The epitopes of the purified clonal mAbs were further confirmed by retesting them on the whole PepSet and on individual peptides of interest that were separately synthesised and purified (GL Biochem, Shanghai, China).

**Figure 6:**
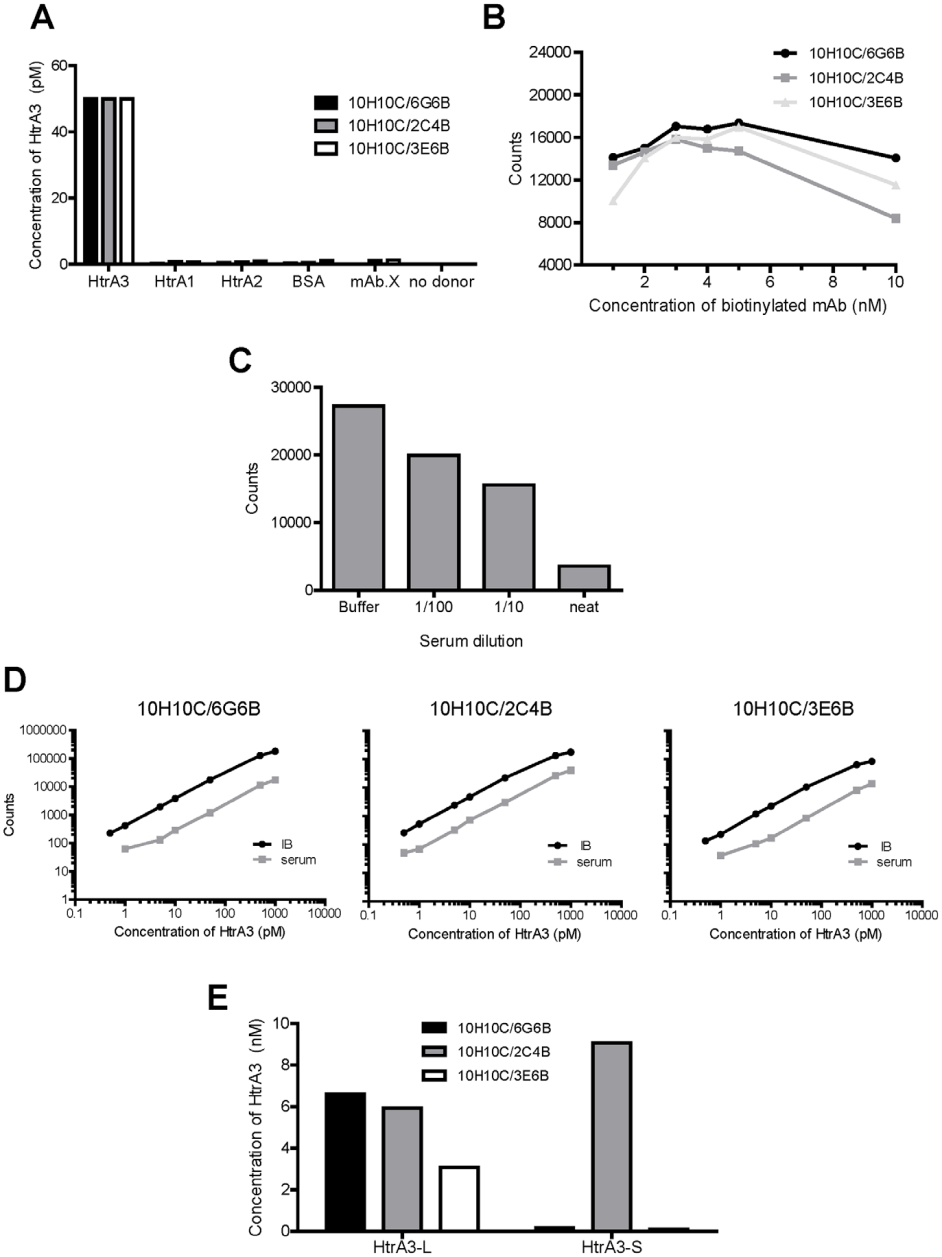
Optimisation and validation of HtrA3 AlphaLISAs. Data is shown for three independent AlphaLISAs employing different mAb pairs [denoted as conjugated (C)/biotinylated (B)]: 10H10C/6G6B, 10H10C/2C4B and 10H10C/3E6B**.** Assays were optimised using HtrA3-L-S305A as the standard with the 1 h (A–C) and overnight (D and E) protocols. (**A**) Detection of an equal amount (50 pM) of HtrA3, HtrA2, HtrA1 or BSA, or measuring HtrA3 (50 pM) when either a biotinylated irrelevant antibody (unrelated mAb) replaced the biotinylated HtrA3 mAb or donor beads were absent. (**B**) Analysis of an equal amount of HtrA3 (50 pM) with various concentrations of biotinylated mAbs to determine the sub-hook point. (**C**) Serum matrix inhibition of the 10H10C/2C4B AlphaLISA. Serum was titrated with an equal amount (500 pM) of HtrA3. (**D**) Representative calibration curves of HtrA3-L-S305A in IB and serum using the overnight protocol. (**E**) Analysis of WT HtrA3 proteins with the overnight AlphaLISA protocol. Conditioned media from HEK-293F cells transfected with WT HtrA3-L or HtrA3-S constructs were assayed, HtrA3 concentrations were determined from a calibration curve of HtrA3-L-S305A standard diluted in transfection medium.

### Human Endometrial Tissues

Human endometrial tissue biopsies from the mid-proliferative (MP) and mid-late secretory (MS) phases of the menstrual cycle were obtained at curettage from women undergoing minor gynaecological surgical procedures, such as laparoscopic sterilisation or tubal patency. Tissues were either snap-frozen in liquid nitrogen and stored at −80°C for protein extraction or fixed in buffered formalin (pH 7.4) and processed to paraffin wax blocks for IHC as previously described [7].

**Table 2:** Accuracy and precision of 10H10C/6G6B and 10H10C/2C4B AlphaLISAs on HtrA3 standard.

		AlphaLISA mAb pairs: Conjugated (C)/Biotinylated (B)										
		10H10C/6G6B					10H10C/2C4B					
Nominal HtrA3Concentration (pM)		Mean pM	% Recovery		Intra-assay % CV	Inter-assay %CV	Mean pM	% Recovery	Intra-assay% CV		Inter-assay %CV	
**In buffer**												
** 1000**		1004.67	100.47	12.43		0.35	1003.22	100.32	11.15		0.33	
** 500**		501.00	100.20	7.72		0.11	500.90	100.18	8.49		0.14	
** 50**		50.12	100.23	8.90		1.93	49.90	99.79	5.38		0.31	
** 10**		11.24	112.35	9.33		5.55	10.47	104.70	6.38		1.63	
** 5**		4.84	96.80	14.52		11.41	5.03	100.64	5.82		2.90	
** 1**		0.64	63.80	10.25		70.75	0.67	67.40	56.70		38.34	
**Average (5–1000 pM)**			102.01	10.58		3.87		101.13	7.44		1.06	
**In serum**												
** 1000**		1002.17	100.22	6.36		0.24	1004.19	100.42	13.28	0.34		
** 500**		500.29	100.06	9.39		0.09	500.43	100.09	6.19	0.16		
** 50**		49.57	99.14	5.84		1.91	48.79	97.58	4.95	4.04		
** 10**		10.60	106.00	13.26		13.77	11.32	113.15	5.27	9.28		
** 5**		4.56	91.24	20.99		17.36	5.17	103.36	14.38	6.04		
** 1**		2.02	201.60	61.40		50.81	1.01	100.75	93.27	79.02		
**Average (5–1000 pM)**			99.33	11.17		6.68		102.92	8.82	3.97		

Concentrations of HtrA3 (HtrA3-L-S305A) were back calculated from the HtrA3 calibration curve and the inter- and intra-assay variations determined using AlphaLISAs in IB and analyte-depleted human serum matrix respectively. Results are from five independent experiments; three assays in triplicate and two assays in ten replicate wells. The intra-assay variation was calculated from one assay of ten replicate wells.

**Table 3:** Limit of detection and lowest limit of quantitation of the HtrA3 AlphaLISAs.

Antibody pair in AlphaLISA	Matrix	LOD (pM)	LLOQ (pM)
**10H10C/6G6B**	**IB**	1.77	5.32
	**Serum**	8.01	24.03
**10H10C/2C4B**	**IB**	0.97	2.90
	**Serum**	1.81	5.44

The limit of detection (LOD) of 10H10C/6G6B and 10H10C/2C4B AlphaLISAs was determined for two matrices (IB and analyte-depleted human serum) from the mean of zero concentration plus three standard deviations in the five assays from Table 2. The lowest limit of quantitation (LLOQ) is three times the LOD.

### Mouse Uterine Tissues

Uterine tissues from non-pregnant WT mice (C57BL/6), and embryo implantation sites from pregnant day (d) 10.5 and d13.5 WT mice or d10.5 HtrA3 null (knockout, KO) mice (C57BL/6 background, generated in-house; unpublished) were obtained as previously described [8]. Tissues were immediately snap-frozen in liquid nitrogen for protein extraction or fixed in formalin for IHC as for the human tissues.

**Table 4:** Reliability and repeatability of the 10H10C/6G6B and 10H10C/2C4B AlphaLISAs on quality control samples.

	AlphaLISA mAb pairs: Conjugated (C)/Biotinylated (B)			
	10H10C/6G6B		10H10C/2C4B	
Amount of HtrA3	Intra-assay % CV	Inter-assay %CV	Intra-assay % CV	Inter-assay %CV
**In buffer**				
**High - 390 pM**	9.80	14.70	8.80	10.61
**Medium - 50 pM**	5.81	10.01	6.73	11.22
**Average CoV %**	7.81	12.35	7.77	10.92
**In serum**				
**High - 590 pM**	8.76	19.82	9.62	16.43
**Medium - 75 pM**	5.76	16.15	8.97	10.90
**Average CoV %**	7.26	17.98	9.29	13.66

The inter- and intra-assay variations of the two HtrA3 AlphaLISAs detecting QCs in IB and serum were determined using a HtrA3-L-S305A calibration curve in IB or analyte-depleted human serum. QCs representing high and medium concentrations of HtrA3 were created in bulk by spiking purified insect-expressed WT HtrA3-L protein in buffer and analyte-depleted human serum matrix respectively and frozen in aliquots. Results are from three independent experiments; two assays in triplicate and one assay in ten replicate wells. The intra-assay variation was calculated from one assay of ten replicate wells.

### Cell Culture and Transfections

The trophoblast HTR-8/SVneo (HTR8) cell line, kindly provided by Dr Charles Graham (Queen’s University, Kingston, ON, Canada) [30], was cultured as previously described [10]. When confluent, cells were trypsinised, pelleted and stored at −80°C until protein extraction.

**Figure 7:**
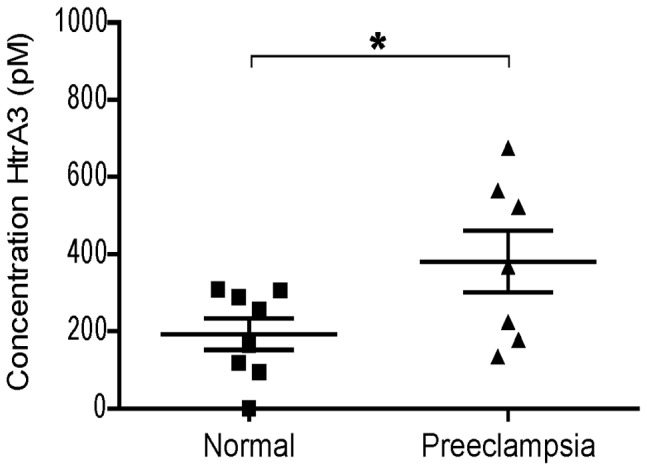
Detection of HtrA3 in serum from normal vs preeclamptic women using the HtrA3 10H10C/2C4B AlphaLISA. Serum HtrA3 concentrations in a cohort of 13–14 week pregnant women who underwent normal pregnancies (Normal, n = 8) or subsequently developed preeclampsia (Preeclampsia, n = 7). Sera were tested using the HtrA3 10H10C/2C4B AlphaLISA with HtrA3-L-S305A standard diluted in serum. HtrA3 levels were significantly higher in preeclamptic women than controls, P<0.04 (*).

**Table 5:** Summary of HtrA3 mAb applications.

		HtrA3 mAb				
Application	Protein source	6G6	9C9	2C4	10H10	3E6
**Western blot**	**rHtrA3**	√	√	√	√	√
	**Human tissues**	√		√	√	√
	**Mouse tissues**			√		√
**Immunohistochemistry**	**Human tissues**	√			√	
	**Mouse tissues**					√
**AlphaLISA** *	**HtrA3-L+S**			10H10C/2C4B	10H10C/2C4B	
	**HtrA3-L**	10H10C/6G6B			10H10C/6G6B	

* For AlphaLISA, the mAb pairing and modifications [either conjugated (C) or biotinylated (B)] are shown.

HEK-293F cells (Life Technologies) were cultured in DMEM (Life Technologies) with 10% FCS. For transient transfection, HEK-293F cells were seeded (8×10^5^ cells/well) in 6-well plates (Nunc) precoated with poly-D-lysine (Sigma) and cultured overnight at 37°C in 5% CO_2_. The following day, cells were transfected with 5 µg of HtrA3 expression constructs [full length human HtrA3-L or HtrA3-S in pcDNA-DEST40 vector (Invitrogen) with a V5 and C-terminal His-tag] or pcDNA empty vector (control transfection), using Lipofectamine 2000 and OPTI-MEM medium (both from Invitrogen) according to the manufacturer’s protocol. Cells were further cultured for 48 hours at 37°C before collection of media for analysis.

### Protein Lysate Preparation from Tissues and Cells

Frozen tissues (from 3 individual human endometrial biopsies or 3 mouse implantation sites) and cell pellets were thawed on ice and lysed into radioimmunoprecipitation assay buffer (65 mM Tris, 1% NP40, 0.25% sodium deoxycholate, 154 mM NaCl, 1 mM EDTA pH 7.4) containing protease inhibitors (Roche, Castle Hill, NSW, Australia). Frozen tissues were freeze/thawed three times (using liquid nitrogen and a 37°C water bath) and ground using ReadyPrep Grinders (Biorad, Hercules, CA, USA) until the mixture was homogenous. DNA was sheared in both tissue lysates and cell pellets by passing the lysate through a 21 g needle at least 15 times using a 1 mL syringe, debris was removed by centrifugation (17, 000 *g*/10 min for tissue lysates and 10, 000 *g*/5 min for cell lysates at 4°C) and the supernatant collected, aliquoted and stored at −20°C until use.

### Western Blotting

Recombinant HtrA1, HtrA2 and HtrA3-L-S305A protein (50 ng), HTR8 cell lysates (50 µg), tissue protein lysates (25 µg), or neat conditioned media (15 µl) from HEK-293F cells (transfected with control, WT HtrA3-L or HtrA3-S constructs), were analysed using standard western blotting (12% reducing SDS-PAGE and PVDF membrane). Protein concentrations were determined using BCA assay kits (Pierce). Primary antibodies, including HtrA3 mAb hybridoma supernatants (1∶1 dilution), purified clonal HtrA3 mAbs (50 µg/mL final concentration), anti-HtrA1 (sc-15465) and anti-HtrA2 (sc-15467) antibodies (400 ng/mL final concentration, both are affinity-purified goat polyclonal, Santa Cruz Biotechnology, Santa Cruz, CA, USA), were incubated overnight at 4°C and probed with horse radish peroxidase (HRP) secondary conjugates, rabbit anti-mouse IgG-HRP (1∶5000, Cell Signalling, Beverly, MA, USA) or rabbit anti-goat IgG-HRP (1∶4000, DAKO, Carpinteria, CA, USA) for 1 h at room temperature. Proteins were visualised by enhanced chemiluminescence (ECl) using Pierce ECl Western Blotting Substrate (Thermo Fisher Scientific, Rockford, IL, USA) and Amersham Hyperfilm ECl film (GE Healthcare, Buckinghamshire, UK). To confirm protein loading, membranes were stripped with Reblot Plus Strong (Millipore, Temecula, CA, USA) according to manufacturer’s instructions and probed with anti-β-actin-HRP antibody (1∶2000, Cell Signalling).

### Immunohistochemistry

Tissue sections (5 µm) were subjected to standard IHC [7], using HtrA3 mAbs or non-immune mouse IgG (Dako) as primary antibodies (8 µg/ml final concentration). For human tissues, sections from 3 individual women for each phase were examined. Biotinylated horse anti-mouse IgG antibody (1∶200, Vector Laboratories, Burlingame, CA, USA) was used as the secondary antibody in conjunction with the Vectastain® Elite ABC kit (Vector). For mouse tissues, sections from 3 individual mice for each genotype were analysed and the Envision® Plus System HRP labelled polymer (anti-mouse, Dako) was used after the primary antibody step. Antigen was retrieved by sequentially microwaving the sections 2×5 min in 10 mM citrate buffer (pH 6.0) for human sections or in 1 mM EDTA (pH 8.0, containing 0.05% Tween 20) for mouse sections. Positive immunostaining (brown precipitate) was revealed by the application of the peroxidase substrate 3,3′-diaminobenzide (DAB) using liquid DAB Chromogen System (Dako). All sections were counterstained with Harris hematoxylin and analysed under an Olympus BH2 microscope fitted with a Fujix HC-2000 high-resolution digital camera (Fujix, Tokyo, Japan).

### Serum from Pregnant Women

Maternal serum samples from healthy pregnant women were retrieved from a blood bank collected between 1999 and 2001 from normotensive women at 13–14 weeks gestation as published [22]. The preeclamptic group (n = 7) developed term (>37 weeks) or preterm (<37 weeks) preeclampsia later in their pregnancy. The controls (n = 8) were matched for maternal age, gestation, parity and smoking status. Preeclampsia diagnosis was based on blood pressure elevation (systolic ≥140 or diastolic ≥90 mm Hg) and new-onset proteinuria (≥2 on dipstick or urinary protein to creatinine ratio >0.3) [31]. The clinical characteristics of both sample groups are listed in Table 1.

### Development and Validation of HtrA3 AlphaLISAs

#### Conjugation of mAbs to acceptor beads

Acceptor beads (Perkin Elmer, Waltham, MA, USA) were conjugated according to manufacturer’s instructions. Briefly, for small scale testing, 0.1 mg acceptor beads and 10 µg mAb (10∶1 ratio) were mixed with 130 mM phosphate buffer (pH 8.0) containing 0.06% Tween-20 in a final volume of 20 µL and conjugated with the addition of freshly prepared NaBH_3_CN (final concentration 1.25 mg/mL). For large scale preparations, 1 mg acceptor beads was mixed with 100 µg mAb in a final volume of 200 µL. The mixture was incubated for 48 h at 37°C on an orbital shaker, the unreacted sites were blocked with 3.25 mg/mL carboxymethoxylamine for 1 h, and the conjugated beads were pelleted in a microcentrifuge (17,000 *g/*15 min) at 4°C. The beads were washed twice with cold Tris buffer (100 mM, pH 8.0), pelleted (17,000 *g/*15 min) at 4°C, resuspended to 5 mg/mL in ice-cold PBS buffer containing 0.05% Proclin-300 (storage buffer), vortexed and sonicated by pulsing for 20 sec with 1 sec intervals at 30% amplitude (Vibracell; Danbury CT, USA). Conjugated beads were stored at 4°C in a screw capped tube.

#### Biotinylation of HtrA3 mAbs

HtrA3 mAbs were biotinylated according to manufacturer’s instructions. Briefly, 100 µg mAb was incubated with 93.7 µM Chromalink Biotin 354S (Solulink, San Diego, CA, USA,) in 200 µl PBS (pH 7.4) for 2 h at room temperature on a rotating wheel. Free biotin was removed with Zeba Spin Desalting Columns (Thermo Scientific) with PBS buffer exchange. The concentration of biotinylated antibody, molecule substitution ratio (molecules of biotin per antibody) and antibody recovery were determined by the absorbance at 280, 354 and 450 nm with PBS as the blank. After the adjustment of the final concentration to 500 nM with PBS/0.1% Tween-20 and 0.05% Proclin-300, the biotinylated antibody was stored at 4°C.

#### Human serum matrix free of HtrA3

To deplete endogenous HtrA3 in serum, 10 µL streptavidin Sepharose beads (Cell Signalling) per mL of serum (pool of 3 individual samples free of known infection, obtained from The Australian Red Cross) were washed in 5 volumes PBS by vortexing and centrifugation (16,000 *g*/5 min) in a microcentrifuge. The beads were mixed with biotinylated HtrA3 10H10 (10H10B) mAb (625 fmole biotinylated mAb/µL beads) in 20 volumes of PBS/beads on a rotating wheel at room temperature for 2 h, pelleted (16,000 *g*/5 min) in a microcentrifuge and washed once with 5 volumes of PBS/beads. The 10H10B-bound beads were resuspended in the serum to be depleted and incubated at 4°C overnight on a rotating wheel. The mixture was centrifuged (16,000 *g*/5 min) in a microcentrifuge and the HtrA3-depleted serum was stored at −20°C.

#### AlphaLISA

AlphaLISA assays were performed in 384 well microplates (Alphaplates, Perkin Elmer) with each sample in triplicates according to manufacturer’s instructions. HtrA3-L-S305A was used as the HtrA3 standard throughout. Briefly, 5 µL of analyte (sample or standard) was first added to wells, 10 µl of 10 nM biotinylated mAb (2 nM final) and 10 µL of 50 µg/mL mAb-conjugated AlphaLISA Acceptor beads (10 µg/mL final) were then sequentially added and the plates sealed with Top Seal (Perkin Elmer). For the 1 h protocol, the plates were incubated at room temperature (22–23°C) for 1 h on an orbital plate shaker (300 rpm). For the overnight protocol, the incubation was at 4°C overnight (16–19 hours). After incubation, 25 µL streptavidin-Donor beads solution at 80 µg/mL (prepared in the dark) was added to each well (40 µg/mL final) and incubated in the dark for 30 min at room temperature on a plate shaker (300 rpm). The europium luminescence was recorded on an Alphascreen compatible reader (Wallac EnVision Multilabel Plate reader equipped with the AlphaScreen module, Perkin Elmer). Data was imported into Masterplex Readerfit software (Hitachi Solutions America, South San Francisco, CA, USA). The HtrA3 concentrations of unknowns were determined from a standard calibration curve fitted using four parameter logistic (4PL) non-linear regression from the above-background counts of triplicate wells.

To select the best antibody combinations, all five HtrA3 mAbs were tested on a constant 50 pM HtrA3 standard. The best combinations (highest signal to background ratio) were selected for further optimisation. The antibody combinations were tested in three different assay buffers (Immunoassay (IB), Universal and HiBlock buffers, all from Perkin Elmer), to determine optimum signal to background ratios. The optimal biotinylated antibody concentration was determined by assaying various concentrations (10, 5, 4, 3, 2, 1 and 0.5 nM final) of the biotinylated antibody on a constant 50 pM HtrA3 standard. The average above-background counts of triplicates were plotted against the biotinylated antibody concentration and the peak (hook) point on the curve was identified; the optimal antibody concentration was determined as the pre-hook (sub-hook) point concentration. The assay was further optimised using HtrA3-L-S305A as the standards diluted from 0 to 1000 pM (1000, 500, 50, 10, 5, 1, 0.5, 0.05 and 0 pM) in IB by lengthening the primary incubation from 1 h at room temperature to overnight at 4°C. The assays were then optimised to detect HtrA3 in human serum by diluting HtrA3 standards in HtrA3-depleted human serum.

The optimised protocols were tested for assay accuracy, reliability and repeatability. Quality control (QC) samples were prepared by spiking purified insect-cell expressed WT HtrA3-L in IB or HtrA3 depleted serum. For each assay, 5 µL of HtrA3 standards or QC samples diluted in buffer or HtrA3-depleted serum were assayed with the overnight protocol. The percentage recovery (accuracy) of the standards in the calibration curve was determined by comparing the back-calculated concentration of standards to the nominal input concentration.

The intra-assay variation (reliability) for both the calibration curve and QCs was calculated from one assay of 10 replicates and the inter-assay variation (repeatability) from five (for calibration curve) or three (for QCs) independent assays performed at least in triplicate on separate days. The limit of detection (LOD) for each assay in each matrix was determined from the mean of zero concentrations plus three times the standard deviation (SD) from the five assays used for the calibration curves. When the zero concentration was beyond the parameters of the 4PL equation, the lowest calculated value in the calibration curve was used. The lowest limit of quantification (LLOQ) was calculated as three times the LOD [32].

To detect HtrA3 in conditioned cell media, 5 µL HtrA3 standards diluted (1–1000 pM) in the transfection medium were assayed together with 5 µL of conditioned media from cells transfected with HtrA3-L or HtrA3-S constructs using the overnight protocol. To detect HtrA3 in pregnant human serum, 5 µL HtrA3 standards prepared in HtrA3-depleted serum were assayed together with 5 µL human serum samples using the overnight protocol. Original concentrations of HtrA3 in the unknown samples were calculated by multiplying the concentration calculated from the calibration curve by the assay dilution factor (×10). Unpaired two-tailed t-test was performed (Prism v 5.03, GraphPad Software Inc., San Diego, CA, USA) for statistical analysis of preeclampsia versus control samples. The correlation (rho [r] with P value) between western blot data and AlphaLISA data was assessed with Spearman’s rank correlation test.

## Results

### 

#### Generation of HtrA3 mAbs

HtrA3-L and HtrA3-S contain identical domains, with the exception of the C-terminal PDZ domain absent in HtrA3-S (Figure 1). Wild type HtrA3 proteins, like other proteases, are susceptible to auto-cleavage, difficult to purify in large amounts and unstable during long-term storage [29]. Substituting the serine with alanine (S305A) in the catalytic site abolishes protease activity and stabilises HtrA3 [13], [15], [29]. This single substitution mutant (HtrA3-L-S305A) with a glutathione S-transferase tag has previously been readily synthesised using a wheat-germ cell-free translation system [29]. HtrA3-L-S305A with a His-tag was synthesised and purified in this study.

To produce HtrA3 mAbs, mice were immunised against HtrA3-L-S305A or a synthetic peptide corresponding to aa 230–239 of both HtrA3-L and HtrA3-S. This led to the cloning and purification of five distinct HtrA3 mAbs: 6G6-17-10-2 (6G6), 9C9-9-10 (9C9), 2C4-1-8 (2C4), 10H10-22-29-6 (10H10) and 3E6-3-32 (3E6) respectively (Figure 1). Four (6G6, 9C9, 10H10 and 3E6) of these were generated from immunisation against HtrA3-L-S305A and one (2C4) against the peptide. 6G6 was isotyped as IgG2b and the other four IgG1. The linear epitope(s) of the four HtrA3 protein derived mAbs were mapped to distinctive regions on the protein (Figure 1).

#### Confirmation of HtrA3 mAb specificity on recombinant human HtrA proteins

The HtrA3 mAbs were tested using western blotting on recombinant human HtrA family members HtrA1-3, where HtrA3-L-S305A was used as HtrA3 (Figure 2A). All mAbs detected HtrA3 at approximately 48 kDa (46.9 kDa plus 1 kDa for the His tag) and not HtrA1 or HtrA2, confirming their high specificity to HtrA3. The integrity of rHtrA1 and rHtrA2 was validated using anti-HtrA1 and anti HtrA2 antibodies respectively (Figure 2A).

To confirm that the mAbs recognise WT HtrA3, HEK-293F cells were transiently transfected with HtrA3-L or HtrA3-S constructs (containing a C-terminal V5 and His-tag, adding 2.4 kDa to the protein size), or control plasmids, and conditioned media was analysed using western blotting (Figure 2B). Human HtrA3 without the signal peptide is predicted to be 46.9 kDa (HtrA3-L; UniprotKB**/**Swiss-Prot: HTRA3_HUMAN_P83110), and 36.3 kDa (HtrA3-S; UniprotKB**/**Swiss-Prot: HTRA3_HUMAN_P83110-2) [33], [34]. Although the exact cleavage sites have not been characterised, both HtrA3-L and HtrA3-S undergo auto-cleavage, removing the Mac25 region to produce ∼33.5 kDa (cleaved form of HtrA3-L, HtrA3-L/C) and ∼23 kDa (cleaved form of HtrA3-S, HtrA3-S/C) products respectively. The cleaved sizes were predicted based on cleavage at aa 144 in the Kazal to protease linker [16]. While 9C9, 2C4 and 10H10 detected both the predicted full length HtrA3-L and HtrA3-S as well as their cleaved products HtrA3-L/C and HtrA3-S/C respectively, 6G6 and 3E6 recognised HtrA3-L and HtrA3-L/C only, consistent with their epitope(s) containing HtrA3-L-specific sequences (Figure 1). The major bands for HtrA3-L (50 kDa) and HtrA3-S (39 kDa) are consistent with their predicted full length sizes (including the V5 and His tag of approximately 2.42 kDa), the smaller minor bands at 36 kDa and 26 kDa represent the predicted cleaved forms of HtrA3. With the exception of 9C9 detecting a non-specific ∼18 kDa band, the other four mAbs were highly specific to HtrA3 as no bands were seen in the control transfection.

Our analysis confirmed that all HtrA3 antibodies, either against HtrA3-L-S305A or the peptide, recognise WT human HtrA3 proteins in an isoform-specific manner.

### Application of HtrA3 mAbs in Western Blot Analysis of Human and Mouse Tissues

The five HtrA3 mAbs were next tested using western blotting on endogenous HtrA3 proteins in human or mouse tissues. For human samples, protein lysates from placental cell line HTR8 and human endometrial tissues of the mid-proliferative (MP) and mid-late secretory (MS) phases of the menstrual cycle were analysed. While 9C9 did not recognise HtrA3 specifically in these samples (data not shown), the other four mAbs (6G6, 2C4, 10H10 and 3E6) detected different isoforms/cleaved forms of HtrA3 proteins (Figure 3A) with higher levels in the MS compared to MP phase, consistent with previous findings [7]. All forms of HtrA3 were detected in the trophoblast cell line, HTR8, with different intensities.

As mouse and human HtrA3 are 95% homologous [5], [6], the mAbs were also tested on mouse uterine tissue lysates. Of the five mAbs, only 2C4 and 3E6 recognised a major specific band corresponding to HtrA3-L [predicted to be 47.4 kDa without the signal peptide (UniprotKB**/**Swiss-Prot: HTRA3_MOUSE_Q9D236) [34]], and a minor band corresponding to HtrA3-L/C (predicted to be 34.3 kDa) in the implantation site lysate (Figure 3B), consistent with HtrA3-L being predominantly expressed in mice [6].

These results demonstrate that the five HtrA3 mAbs recognise HtrA3 proteins in the human versus mouse with different specificities.

### Application of HtrA3 mAbs in IHC on Human and Mouse Tissues

All five mAbs were tested using IHC on formalin-fixed human and mouse uterine tissues. While 10H10 and 6G6 specifically stained the luminal and glandular epithelium in human endometrial tissues with a higher level in the MS than MP phase (Figure 4 A–D), only 3E6 specifically stained HtrA3 in the mouse implantation sites with a higher level on d10.5 than d13.5 (Figure 4 E–F), consistent with previously observed patterns using a HtrA3 specific sheep antibody [8]. These results further demonstrate that the five HtrA3 mAbs have different specificities towards human relative to mouse HtrA3 proteins.

### Development of High-throughput, Specific and Sensitive HtrA3 AlphaLISA Assays

#### Selection of the best mAb pairs for HtrA3 AlphaLISA

To measure HtrA3 levels in biological fluids, the HtrA3 mAbs were incorporated into an AlphaLISA (Figure 5). As AlphaLISA requires two distinct antibodies, one conjugated to acceptor beads and the other biotinylated, all five mAbs were separately conjugated on a small scale or biotinylated and all possible 20 (5×4) combinations tested in IB. The best combinations were when 10H10 was conjugated to the acceptor beads (10H10C) and paired with any of the other four biotinylated mAbs, with a signal to background ratio 6–30 times higher than any other combinations (data not shown). When 10H10C was paired with a biotinylated mAb (×B), the best pairings were in the order of 6G6B>2C4B>3E6B>9C9B (data not shown). The best three pairs, 10H10C/6G6B, 10H10C/2C4B and 10H10C/3E6B, were further tested.

### HtrA3 AlphaLISA Specificity

All three HtrA3 AlphaLISAs (10H10C/6G6B, 10H10C/2C4B and 10H10C/3E6B) were specific to HtrA3 and did not detect HtrA1, HtrA2 or BSA (Figure 6A). HtrA3 was not detected when the donor beads were omitted or when an unrelated biotinylated mAb was substituted for the HtrA3 biotinylated mAb (Figure 6A).

### Optimisation of HtrA3 AlphaLISA Assay for Serum Detection

To determine the optimal concentration of the biotinylated antibody in the AlphaLISAs, 50 pM of HtrA3 standard was assayed in IB with various concentrations of biotinylated antibodies (Figure 6B). All three antibody pairs responded in a similar fashion and the sub-hook point was 2 nM for three assays.

We next tested the matrix effect of serum in the assay. Spiking 5 µL neat serum (representing 10% of the total assay volume) in the 10H10C/2C4B AlphaLISA with 500 pM HtrA3 standard decreased the signal 7.6 fold (Figure 6C). In general, the signal was inversely proportional to the amount of serum in the assay. This highlighted the need to construct HtrA3 standard curves in HtrA3-depleted serum when examining serum.

To further increase the assay signal, extending the incubation of the analyte, 10H10C acceptor beads and biotinylated antibodies in IB from 1 h to overnight doubled the signal (data not shown). The overnight protocol was employed for subsequent studies.

### HtrA3 Calibration Curves

To account for the serum matrix effect, the HtrA3 standard (HtrA3-L-S305A) was diluted in IB or HtrA3-depleted serum respectively, and calibration curves in IB and serum for all three antibody pairs were constructed using optimised conditions (Figure 6D). The curves were linear between 1–1000 pM for all three assays in both matrices (Figure 6D). For each HtrA3 concentration and for all three mAb pairs, the signal was 5–11 fold lower in serum than in IB (Figure 6D).

### Testing HtrA3 AlphaLISAs on WT HtrA3 Proteins

We next tested whether the three AlphaLISAs could recognise WT HtrA3 in the media of transfected cells. The 10H10C/2C4B AlphaLISA detected both HtrA3-L and HtrA3-S, whereas the 10H10C/6G6B and 10H10C/3E6B AlphaLISAs recognised HtrA3-L only (Figure 6E), consistent with 6G6 and 3E6 being HtrA3-L specific (Figure 2B). Both 10H10C/2C4B and 10H10C/6G6B detected similar concentrations of HtrA3-L, however, the 10H10C/3E6B AlphaLISA detected a lower concentration (Figure 6E). Accordingly, the 10H10C/6G6B AlphaLISA was selected to detect the HtrA3-L isoform and the 10H10C/2C4B AlphaLISA to detect both HtrA3 isoforms.

### HtrA3 AlphaLISA Working Range and Intra- and Inter-assay Variations

The accuracy and precision of the 10H10C/6G6B and 10H10C/2C4B AlphaLISAs were further determined using calibration curves in IB or serum (Table 2). The average recovery percentages between 5–1000 pM were highly accurate as the back calculated concentrations were within 3% of the nominal concentrations. The calibration curve was also precise with average intra-assay variations of 7–11%. Overall, assays in serum matrix showed higher variability than in IB, and the 10H10C/6G6CB assay had higher variability than 10H10C/2C4B for both IB and serum. The estimation of concentration was also very reproducible within this range as reflected by the low inter-assay variation (1–4%). The intra-assay variation was higher than the inter-assay variation. When the lowest limit of quantification was calculated (Table 3), the lower limit of 5 pM was acceptable for all except 10H10C/6G6B in serum which has a calculated LLOQ at 24.03 pM. Thus, in serum, the working ranges are 24–1000 pM (1176–49000 pg/mL) for 10H10C/6G6B and 5–1000 pM (245–49000 pg/mL) for 10H10C/2C4B. The upper limit of quantitation was not determined.

The two assays were further tested on active recombinant HtrA3 spiked in IB or serum as QC samples. The assays were reliable with intra-assay variation ranging between 5.81%–9.80%, and repeatable with inter-assay variations ranging between 10.01%–19.82% (Table 4).

### Application of HtrA3 AlphaLISA to Detect Preeclampsia in Early Pregnancy

The validity of the 10H10C/2C4B AlphaLISA detecting all forms of HtrA3 was further confirmed by testing a previously published cohort of sera from pregnant women at 13–14 weeks of gestation [22]. Using western blotting, serum HtrA3 levels at this gestation were found to be significantly higher in women who subsequently developed preeclampsia (Preeclampsia, n = 8) than those who had normal pregnancies (Normal, n = 8) [22]. The AlphaLISA detected a similar difference (P<0.04) between the Preeclampsia group (n = 7, as one sample was exhausted) and Normals (n = 8) (Figure 7). Spearman’s rank correlation analysis showed a significantly positive correlation between the western blot and AlphaLISA data (r = 0.539, P<0.038). This cohort was also tested using the long form specific 10H10C/6G6B AlphaLISA (data not shown) suggesting that it is the long form of HtrA3 that is predominantly present in serum.

The various applications of the five HtrA3 mAbs in mouse and human are summarised in Table 5.

## Discussion

We have generated and cloned five HtrA3 mAbs (6G6, 9C9, 2C4, 10H10 and 3E6) with unique epitopes, three (9C9, 2C4 and 10H10) recognise both HtrA3-L and HtrA3-S isoforms and their cleaved products, and two (6G6 and 3E6) detect HtrA3-L and HtrA3-L/C only. These mAbs are highly specific to HtrA3 and do not recognise human HtrA1 or HtrA2. The antibodies are confirmed to be applicable in western blotting and IHC analysis of endogenous HtrA3 proteins in the mouse and human tissues. We have further established and validated two HtrA3 AlphaLISA assays using two different antibody pairs, one detecting all HtrA3-L and HtrA3-S isoforms (10H10C/2C4B) and one detecting HtrA3-L isoforms only (10H10C/6G6B). These assays are highly specific to HtrA3 and suitable for large scale screening of human serum with a lower limit of quantitation of 5–24 pM and 7–18% intra- and inter-assay variations in quality controls. The 10H10C/2C4B AlphaLISA recognising all HtrA3 isoforms detects significantly higher levels of HtrA3 in serum at early gestation in pregnant women who subsequently developed preeclampsia than controls as previously published [22]. These HtrA3 mAbs provide critical tools for the diagnosis of preeclampsia and possibly other human diseases associated with HtrA3 dysregulation, and will enable further characterisation of HtrA3 isoform-specific biology.

The five HtrA3 mAbs were first validated using western blotting for specificity using recombinant HtrA3 proteins, and then evaluated on endogenous HtrA3 in human and mouse tissues using western blotting and IHC. In western blot analysis, four mAbs (6G6, 2C4, 10H10 and 3E6) detected endogenous proteins in human tissues and two (2C4 and 3E6) in mouse tissues, and the band patterns were similar to that of previously published sheep polyclonal HtrA3 antibodies [7], [8]. In IHC, two mAbs (6G6 and 10H10) specifically immunostained HtrA3 in human endometrial tissues and one (3E6) recognised HtrA3 in mouse tissues; the staining was highly similar to IHC using the sheep antibodies [7], [8]. Thus mouse and human specific antibodies for different applications were identified. This in turn highlighted that not all five HtrA3 mAbs were suitable in all applications and for all species, suggesting that human and mouse HtrA3 proteins differ in their epitope formation despite high sequence similarities.

Three of the HtrA3 mAbs were used to develop AlphaLISAs that are robust, highly sensitive and suitable for high-throughput screening of HtrA3 isoforms in serum at picomolar levels across at least three orders of magnitude. In AlphaLISA, the antibody pair 10H10C/2C4B detected both HtrA3-L and HtrA3-S isoforms, whereas 10H10C/6G6B recognised HtrA3-L only. Importantly, these HtrA3 AlphaLISA assays only required a small volume (5 µL) of serum and were optimal for analysing undiluted serum with LLOQs and intra- and inter-assay variations comparable to other homogenous assays and Alphascreens [35]–[38]. Notably, the assay for both HtrA3 isoforms detected significantly higher levels of HtrA3 at 13–14 weeks of gestation in pregnant women who subsequently developed preeclampsia later in gestation, compared with women who had a normal pregnancy. The concentrations detected in the original human pregnant serum ranged from 118–308 pM (5782–15,092 pg/mL) in normotensive women and 135–765 pM (6615–37,485 pg/mL) in women who developed preeclampsia. However, as the samples were from a cohort that had been used previously (thawed multiple times) they were sub-optimal. Future studies will utilise HtrA3 AlphaLISAs to screen a large number of appropriate serum samples to establish the robustness of HtrA3 as an early marker for preeclampsia. Our current studies suggest that the long isoform of HtrA3 is the predominant form present in the sera, however further studies are required to clarify this.

Earlier detection of preeclampsia would greatly facilitate careful monitoring and supportive therapies to reduce morbidity and mortalities in both the mother and baby. Currently there are no clinical assays with a high enough positive predictive value for early detection of preeclampsia. Financially, advanced notice prior to the development of preeclampsia would reduce the overall costs involved in healthcare by strategic focussing on high risk women rather than all pregnant women [39].

The causes of preeclampsia may be multi-factorial and thus combining a number of markers would be beneficial to diagnose preeclampsia. There is the potential of combining one or several markers such as soluble endoglin, soluble fms-like tyrosine kinase 1, placental growth factor, cell free DNA/RNA, placental protein 13 (PP13), pentraxin 3 and pregnancy associated plasma protein A, with the biophysical marker Doppler uterine artery ultrasound and predisposing maternal risk factors [23], [40], [41]. It is possible that combining HtrA3 detection with other markers may strengthen the power of preeclampsia detection.

Future studies will also investigate the potential of these HtrA3 mAbs in modulating the proteolytic activity of HtrA3. A neutralising mAb would have implications for therapeutic opportunities in diseases where HtrA3 is abnormally higher than normal such as preeclampsia.

In summary, HtrA3 mAbs developed in this study will enable thorough investigations into the mechanisms of isoform-specific actions of HtrA3 in ovary development, placental development and cancer progression, and provide a means to characterise the effects of dysregulation of HtrA3 in these processes. These mAbs will also broaden the development of other immunoassays where dysregulation of HtrA3 is a disease marker. The newly developed HtrA3 AlphaLISA assays are suitable for large scale screening of human fluids and have been validated on human serum.
